# Simulation of Capacitorless DRAM Based on the Polycrystalline Silicon Nanotube Structure with Multiple Grain Boundaries

**DOI:** 10.3390/nano13132026

**Published:** 2023-07-07

**Authors:** Jin Park, Sang-Ho Lee, Ga-Eon Kang, Jun-Hyeok Heo, So-Ra Jeon, Min-Seok Kim, Seung-Ji Bae, Jeong-Woo Hong, Jae-won Jang, Jin-Hyuk Bae, Sin-Hyung Lee, In-Man Kang

**Affiliations:** School of Electronic and Electrical Engineering, Kyungpook National University, Daegu 702-201, Republic of Korea

**Keywords:** polycrystalline silicon, one-transistor dynamic random-access memory, grain boundary, nanotube, metal–oxide–semiconductor field-effect transistor, dual-gate, statistical analysis

## Abstract

In this study, a capacitorless one-transistor dynamic random-access memory (1T-DRAM), based on polycrystalline silicon (poly-Si) nanotube structure with a grain boundary (GB), is designed and analyzed using technology computer-aided design (TCAD) simulation. In the proposed 1T-DRAM, the 1T-DRAM cell exhibited a sensing margin of 422 μA/μm and a retention time of 213 ms at T = 358 K with a single GB. To investigate the effect of random GBs, it was assumed that the number of GB is seven, and the memory characteristics depending on the location and number of GBs were analyzed. The memory performance rapidly degraded due to Shockley–Read–Hall recombination depending on the location and number of GBs. In the worst case, when the number of GB is 7, the mean of the sensing margin was 194 µA/µm, and the mean of the retention time was 50.4 ms. Compared to a single GB, the mean of the sensing margin and the retention time decreased by 59.7% and 77.4%, respectively.

## 1. Introduction

Currently, dynamic random-access memory (DRAM) is a representative volatile memory consisting of one transistor–one capacitor in one cell. DRAMs are the most used memory device because of their small chip area, high speed, and low process cost [1]. As the demand for conventional 1T-1C DRAM has increased, the chip sizes are being continuously scaled down. However, the channel length of the 1T-1C DRAM is gradually reduced, resulting in electrical performance degradation due to short channel effects. Furthermore, it is difficult to reduce the increase in capacitors while the cell capacitance is increasing. As a result, the capacitor aspect ratio of the conventional 1T-1C DRAM is limited. To solve this problem, one-capacitor DRAMs (1T-DRAM) without a capacitor are attracting attention [2,3,4,5,6,7,8,9,10,11]. The 1T-DRAM consists of one transistor without a capacitor and uses a partially definite floating body area on the Silicon-on-Insulator (SOI) substrate as a data storage region. The 1T-DRAM used excess holes in the storage region to distinguish between states “1” and “0”. A 1T-DRAM has merits such as simple fabrication and a higher chip density, compared with the conventional 1T-1C DRAM. However, a small device size of 1T-DRAM has an increase in recombination/generations at the PN Junction due to the absence of the storage region, which has a small sensing margin and a short retention time. Various of 1T-DRAMs with a dual-gate structure have been studied to overcome the limitations of the retention time [12,13,14,15,16]. In particular, the 1T-DRAM of the gate-all-around-based nanotube structure exhibited not only high on-current but also excellent retention time [16]. The SOI wafer has a complicated fabrication process and a high process cost. In this regard, 1T-DRAM using polycrystalline silicon (poly–Si) is proposed. Because the SOI-like structure fabrication is a low-cost and high-efficiency process, it can be applied to the three-dimensional, vertically stacked and multi-layered structure [17]. However, grain boundaries (GBs) that exist in poly-Si directly affect memory performances. In the case of the n-type MOSFET, as electrons are trapped in the GBs, acceptor-like traps form sharp energy barriers. The induced potential barrier caused by GB not only directly prevented the current flow, but also induced trap-assist tunneling (TAT) and reduced the memory performance, due to recombination with stored holes in the body [18,19]. GBs are randomly generated for each grain, and traps exist in GBs. Considering this, it is assumed that GB exists in the source, body, and drain region and its effects are investigated.

In this study, we proposed the poly-Si based nanotube structure 1T-DRAM with multiple GBs. The optimized 1T-DRAM exhibited a high-sensing margin because of the two independent gates surrounding the channel, thus resulting in an excellent retention time. To investigate the effect of GBs, we analyzed when seven single GBs exists at each location of the source, body, and drain. Also, the sensing margin and the retention time are statistically shown with the 127 samples, considering the number of all cases when seven GBs exist. The proposed device is designed using a two-dimensional technology computer-aided simulation.

## 2. Device Structure and Simulation Methodology

**Figure 1:**
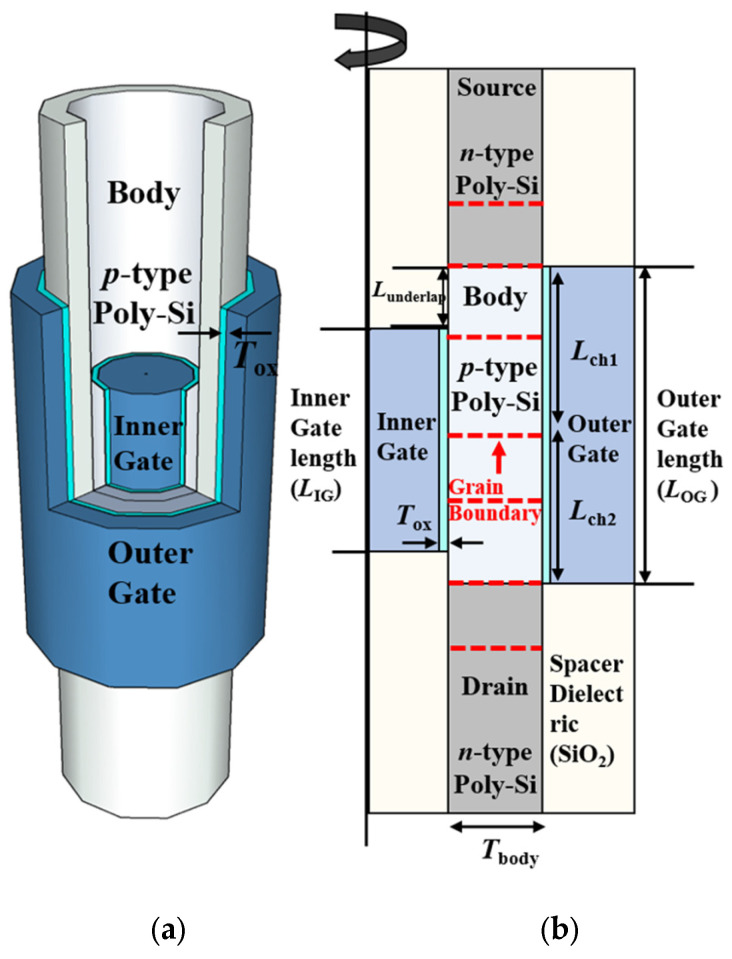
(**a**) Three-dimensional schematic of the proposed nanotube-based 1T-DRAM and (**b**) cross-sectional view with multiple GBs.

Figure 1 shows the three-dimensional view and cross-section view of the proposed 1T-DRAM with seven GBs. The proposed nanotube-based 1T-DRAM shows superior channel controllability as the outer gate and inner gate surrounded the channel. The device parameters of the proposed 1T-DRAM refer in [16]. By applying different voltages to the two gates, the 50 nm outer gate performed the same role as the conventional metal–oxide–semiconductor field-effect transistor (MOSFET) during the read operation, and the 30 nm inner gate controlled the stored hole to perform a memory operation. The work functions of the outer gate and inner gate are 4.8 eV and 5.2 eV, respectively, and the high-work function of the inner gate increased the energy band to create a potential well for hole storage. Additionally, using the underlap to the inner gate, the retention time increased because the gate electric field is reduced at the PN junction [12]. The doping concentration of the source and the drain is n-type, 5 × 10^19^ cm^−3^, and the doping concentration of the body is p-type, 1 × 10^17^ cm^−3^ with the constant doping profile. The geometric parameters are summarized in Table 1. As the gate dielectric, 2 nm of HfO_2_ is used to increase gate controllability, and the dielectric constant of 22 is used [20].

**Figure 2:**
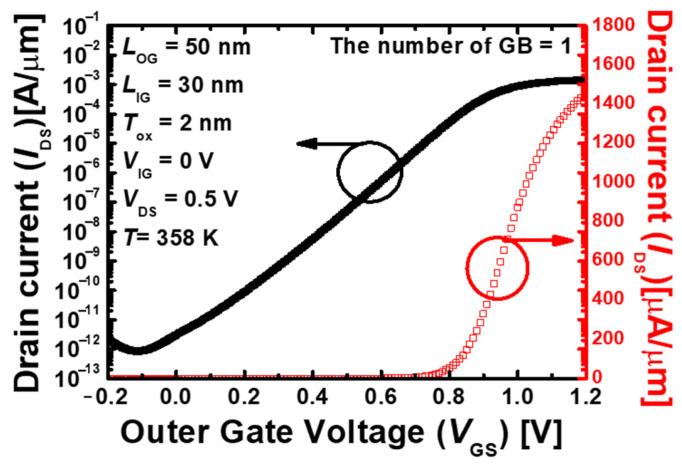
I_d_–V_g_ transfer characteristic of the proposed 1T-DRAM with a single GB.

We assumed that the seven GBs exist at the source, channel, drain, source, and body junctions, and body and drain junctions to consider the effect of GBs. When GB exists, a sharp band peak is formed in the conduction band by the charge trapped in the GB. This prevents the flow of electrons, reducing the conductance of the channel and increases threshold voltage. Also, it degraded the performance of the read operation which is the same as the conventional MOSFET operation. That is, the sensing margin distinguished between the read “1” state and the read “0” state reduced. In addition, GB increases the SRH recombination rate by trap-assisted tunneling (TAT) at the PN junction and leads to decrease the retention time due to recombination of the electron trapped in the GB with the hole stored in the body. The grain size is divided based on the GB existing in the center of the body, and it assumed at the same interval of 12.5 nm considering the total length of the channel. Also, the grain size assumed 20 nm when GBs exist at the source and drain in the n+ doping region. The trap density of the GBs in the proposed 1T-DRAM used data, as shown in Figure 2 of reference [21]. There are four traps in the GB: donor-like shallow trap (DST), donor-like deep trap (DDT), acceptor-like shallow trap (AST), and acceptor-like deep trap (ADT). For the accurate data of the proposed 1T-DTAM, physical models such as the Shockley–Read–Hall (SRH) recombination, the Hurkx trap-assisted tunneling (TAT), the nonlocal band-to-band tunneling (BTBT) model, the Fermi–Dirac statistical model, the doping-dependent model, and the quantum-confinement effect model are applied to the simulation.

## 3. Results and Discussion

To consider the effect of GB present in poly–Si, it is optimized by assuming that GB exists in the center of the body. Figure 2 shows the drain current (I_DS_) vs. gate voltage (V_GS_) of the proposed 1T-DRAM with a single GB in the center of the body. The threshold voltage (Vth) was 0.56 V with the drain current of 10^−7^× (W/L) A/µm. The drain current per μm is normalized to π × (d_core_ + t_si_). The d_core_ is the diameter of the inner gate and t_si_ is the thickness of the nanotube [22,23]. The proposed 1T-DRAM exhibited high on-current (I_on_) because the outer gate and inner gate controlled the body. The outer gate formed an inversion channel that is similar to the conventional MOSFET, and the inner gate formed an accumulation layer and increased mobile carriers. Therefore, the inner gate performs the same role as a dual gate, and a higher current than conventional nanowire devices is obtained [24,25,26].

**Figure 3:**
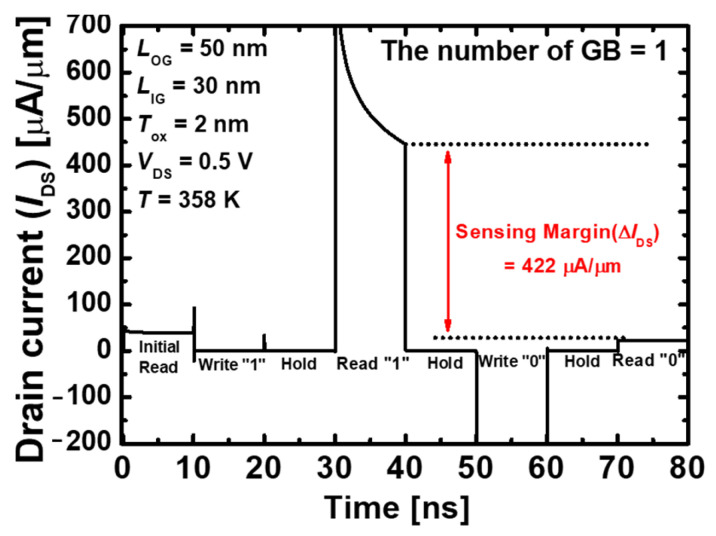
Transient characteristic of the proposed 1T-DRAM with a single GB. The operating time is 10 ns.

Figure 3 shows the transient characteristics of the proposed 1T-DRAM when a single GB exists in the middle of the body. Table 2 summarized the operating bias scheme for the memory performance of the proposed 1T-DRAM. The sensing margin is the difference between the read “1” current and the read “0” current, and the proposed 1T-DRAM obtained an excellent sensing margin of 422 µA/µm.

**Figure 4:**
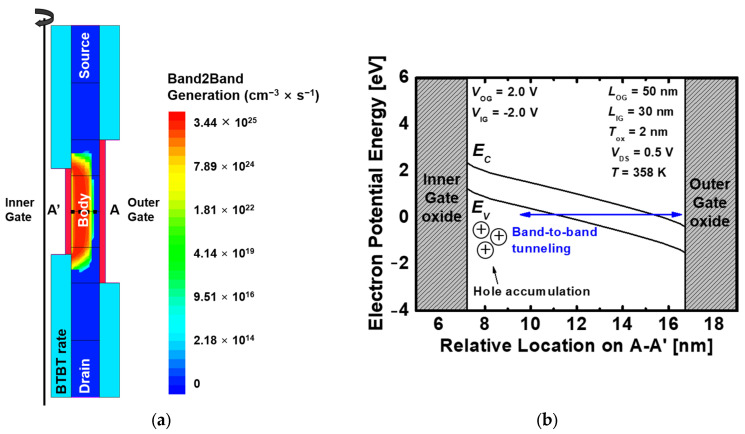
(**a**) Contour map of the BTBT rate of the proposed 1T-DRAM during the program (write “1”) operation and (**b**) Energy band diagram of the proposed 1T-DRAM in the program operation. The energy band is extracted at the center of the body.

Figure 4a shows the write “1” operation of the proposed 1T-DRAM. During the write “1”, it is performed through BTBT between the outer gate and the inner gate. The energy band between the two gates is formed by applying 2.0 V to the outer gate and −2.0 V to the inner gate, as shown in Figure 4b. A tunneling path is formed by the electric field in the same direction, and the excess holes generated through BTBT are accumulated at the inner gate. The accumulated holes are stored by the high-work function and negative voltage of the inner gate.

**Figure 5:**
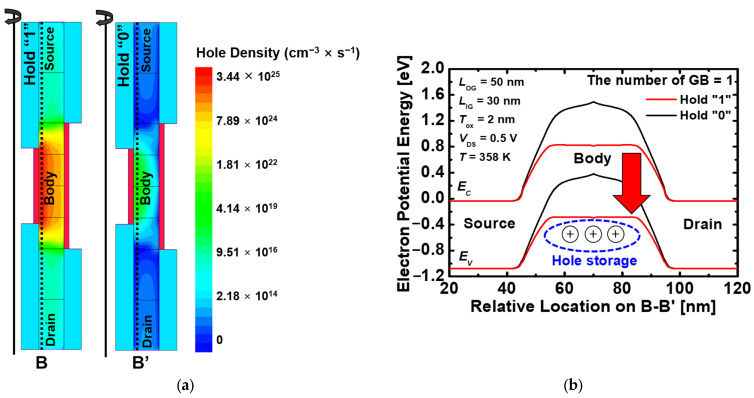
(**a**) Contour map of the hole density of the proposed 1T-DRAM in the hold “1” and the hold “0” and (**b**) Energy band diagram of the proposed 1T-DRAM in the hold operation. The energy band is extracted at 2 nm below the inner gate oxide.

Figure 5b shows the difference in the energy band depending on the hole density during the hold operation. Holes generated during the write “1” operation are stored in the body region by the negative voltage of the inner gate, which is defined as the hold “1” state. Furthermore, the hold “0” state is defined after removing the holes stored in the body by applying a negative bias to the drain. Figure 5b shows the difference in the energy band depending on the hold density during the hold operation. When the hold “1” state, the energy band is reduced compared to the hold “0”, because it has the same effect as applying positive bias by the holes stored in the body.

**Figure 6:**
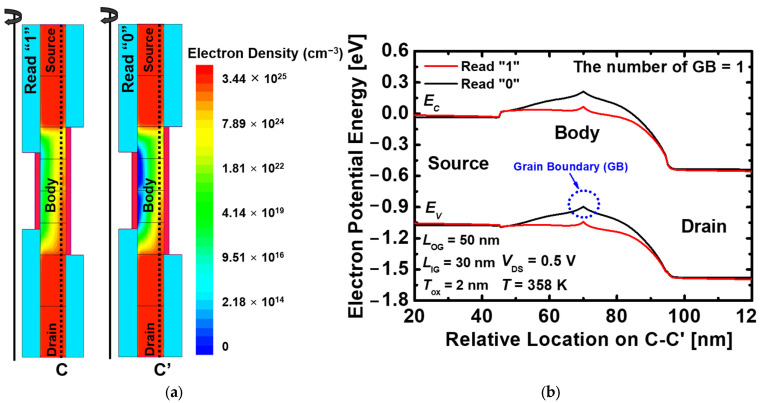
(**a**) Contour map of the electron density of the proposed 1T-DRAM during the read operation and (**b**) Energy band diagram of the proposed 1T-DRAM in read “1” and read “0”. The energy band is extracted at 2 nm below the inner gate oxide.

Figure 6a shows the electron density difference during the read operation of the proposed 1T-DRAM. The electron density is high in the read “1” due to the holes stored in the body. As shown in Figure 6b, the energy band is reduced, and has a higher current flow than the read “0”.

**Figure 7:**
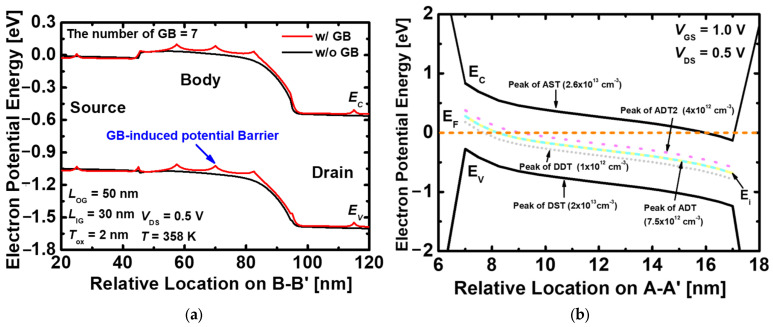
(**a**) Energy band of the proposed 1T-DRAM with and without GBs during the read operation and (**b**) Energy band diagram depending on the trap distribution in the proposed 1T-DRAM.

Figure 7a shows the variation of the energy band depending on with and without GB. When GBs exist, sharp energy band peaks are formed at each location, because electrons are trapped in GB traps, increasing the energy barrier. In addition, the captured electrons induce a repulsive force in their surroundings, resulting in preventing the current flow and degraded memory performance [27]. Figure 7b shows the types of traps that exist in the GB as energy bands along the A-A’ direction in Figure 4a. To investigate only the effect of GB, no voltage is applied to the gate and drain, and the energy band is formed by the difference in work function between the outer gate and the inner gate. In the proposed 1T-DRAM, there are four traps: AST, ADT, DDT, and DST. Among them, DDT and DST are neutral states because they are below the Fermi level and are filled with electrons. The AST is also a neutral state due to being located above the Fermi level, and electrons did not exist in the trap. Therefore, the proposed 1T-DRAM is most affected by the two ADTs. On the other hand, the layer accumulated at the inner gate side is close to the p-type doping concentration and is affected by the DDT. However, the peak value of DDT is small, and has hardly any effect.

**Figure 8:**
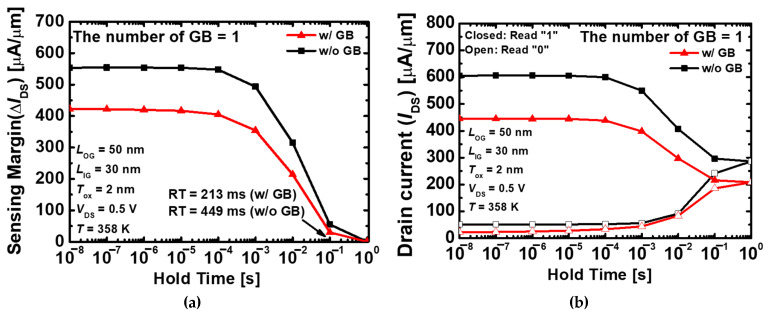
(**a**) Sensing margins as a function of the hold time with depending on with and without GB and (**b**) Variation of the read currents in the “1” and “0” state depending on with and without GB. The closed box is indicate of the drain current variation of the read “1”, and the open box is indicate of the drain current variation of the read “0”. Also, the red line is the variation in drain current when GBs are exist, and the black line is the variation in drain current when GBs are not exist.

Figure 8a shows the memory characteristics depending on with and without GB in the proposed 1T-DRAM. When a single GB exists, the retention time decreased by 2.1 times from 449 ms to 213 ms compared to without GB. The retention time is an important factor among various evaluation indicators of memory devices. This is the minimum time that stored data can be correctly detected without data refresh. The retention time is defined as the hold time when the sensing margin is 20 µA/ µm [28]. Figure 8b shows the drain current variation depending on with and without GB. The 1T-DRAM tends to return to the initial state during the hold time after the program or erase operation. To maintain equilibrium, the SRH recombination rate increased during the read “1” operation, and the generation rate increased during the read “0” operation. When GB do not exist, the read “1” current decreased rate and the read “0” current increased rate are similar, however, when GB exists, the read “1” current decreased more rapidly than the read “0” current.

**Figure 9:**
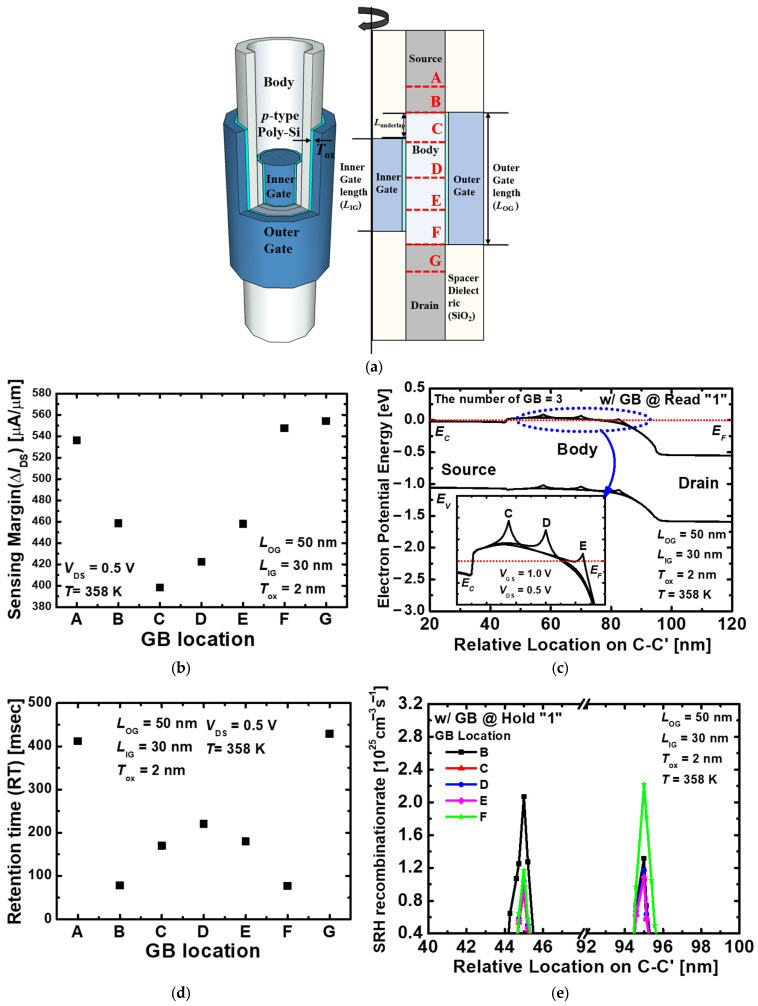
(**a**) Cross-sectional view of the proposed 1T-DRAM depending on the GB location. (**b**) Sensing margin variation depending on the GB location from A to G. (**c**) Energy band variation when GB is located at C, D, and E in the body of the proposed 1T-DRAM. The energy band is extracted at 2 nm below the inner gate oxide. (**d**) Retention time variation depending on the GB location from A to G. (**e**) SRH recombination rate depending on the GB location during the hold “1” state.

Figure 9a shows the cross-section view depending on the location of multiple GBs in the proposed 1T-DRAM. It is assumed that the number of GBs from the source to the drain is seven, the number of GBs in the source and the drain is two, the number of GBs in the body is three, and the number of GBs in the source junction and the drain junction is two. Figure 9b shows the sensing margin depending on each GB location. When a single GB is located at A, F, and G, the sensing margin is similar to when GBs do not exist. When GBs are located at A and G, where the n+ region exists with a high-doping concentration, electrons are filled in the trap and lower the energy barrier of the GBs [29,30]. When the GB exists in F, it is located at the body and the drain junction. However, it is mainly affected by the drain voltage and exhibits a phenomenon similar to drain-induced barrier lowing (DIBL) [31]. Therefore, the energy band is reduced, the effect of GB is neglected and a high-sensing margin is obtained. When the GB is located at B, the source and the body junction, trap-assist tunneling (TAT) occurred and the recombination/generation rate of stored holes increased [32]. However, when B is located near the source, many electrons are injected and slightly affected the reduction of the sensing margin.

Figure 9c shows the energy bands when GBs are located at C, D, and E in the body. When the GB is located close to the drain, the energy band decreased because of the positive drain voltage. The energy barrier by the GB is also lowered, reducing the number of traps that exists in the empty energy states between the Fermi level and the conduction band edge. Therefore, the sensing margin increases when GB is located at E, and the sensing margin is lowest at C. Figure 9d shows the retention time characteristics depending on the GB location. When the GBs are located at A and G, the tendency is the same as the sensing margin, and similar retention times are obtained when the GBs are not present. Also, the retention time is lower when the GB is located at B, and F and increased as the GB became closer to the center of the body. Figure 9e shows the SRH recombination rate during the hold “1” operation when GBs are located from B to F. The SRH recombination rate is high at B and F because it is affected by TAT. During the hold operation, the holes stored by TAT recombine and caused leakage. This leakage increased with the increase in hold time. Therefore, it has a short retention time at the PN junction, and the retention time increases as the distance from the PN junction increases. The sensing margin and retention time depending on GB location are summarized in Table 3.

Table 4 summarized the sensing margin and retention time of 1T-DRAM previously reported. The proposed 1T-DRAM in this paper shows superior memory characteristics compared to other devices.

We considered the effect of random GBs that exist in poly–Si and analyzed its reliability. The sensing margin and retention time for 127 samples in the proposed 1T-DRAM are shown as a histogram. The sample group and sample number of GBs are summarized in Table 5.

**Figure 10:**
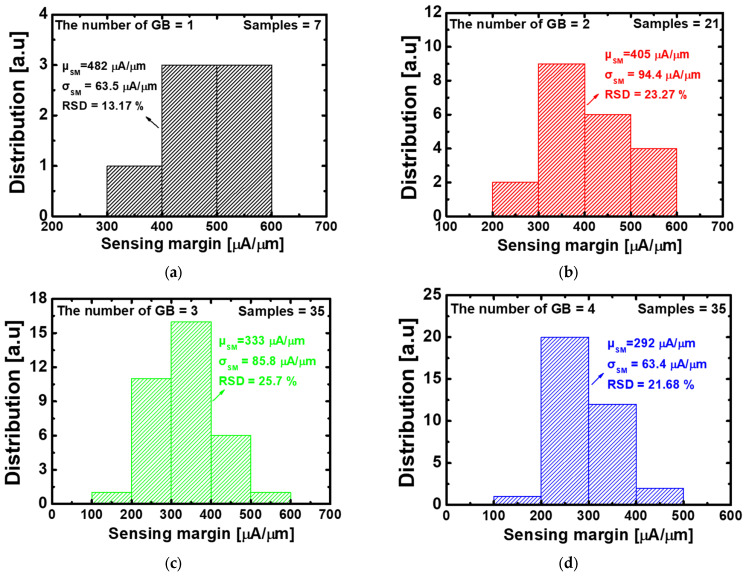

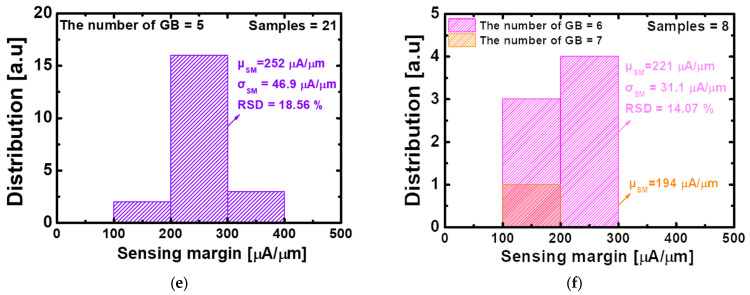
Histograms of sensing margin for 127 samples in the proposed 1T-DRAM (**a**) the number of GB is 1 (**b**) the number of GB is 2 (**c**) the number of GB is 3 (**d**) the number of GB is 4 (**e**) the number of GB is 5, and (**f**) the number of GB is 6 and 7.

Figure 10 shows the histogram of the sensing margin depending on the number of GBs. When the number of GB is seven, the mean decreases from 482 µA/µm to 194 µA/µm, and the standard deviation (SD) of the sample decreased accordingly. When the number of GBs is small, the sensing margin average ranges from 100 μA/μm to 500 μA/μm. However, as the number of GBs increased, the maximum average decreased, and the ranges of the sensing margin decreased from 100 μA/μm to 200 μA/μm. As the average decreased, the standard deviation (SD) also decreased from 63.5% to 31.1%. This is because as the number of GBs increases, the number of sharp band peaks increased in Figure 7a and the memory performance degraded. Nevertheless, it shows a high-sensing margin of over 100 µA/µm (>20 µA/µm at 358 K [28]). In the case of the sensing margin, it is mainly affected when GB is located at B or C or D, or E. The probability that GB exists at B or C or D or E is 4/7 when the number of GB is one, 18/21 when the number of GB is two, and 34/35 when the number of GB is three, respectively. Therefore, the RSD increased because the probability of being included in B or C or D, or E also increased. However, when the number of GBs is four or more, RSD decreases owing to GBs definitely existing at B or C or D, or E.

**Figure 11:**
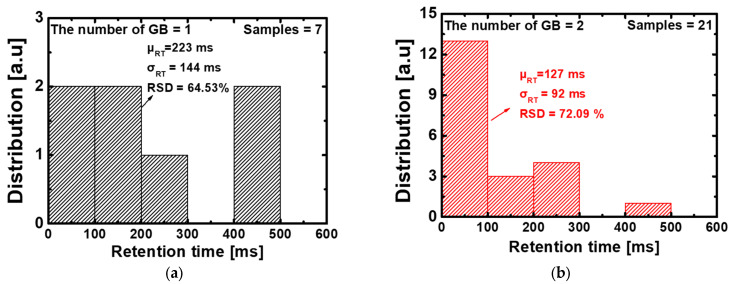

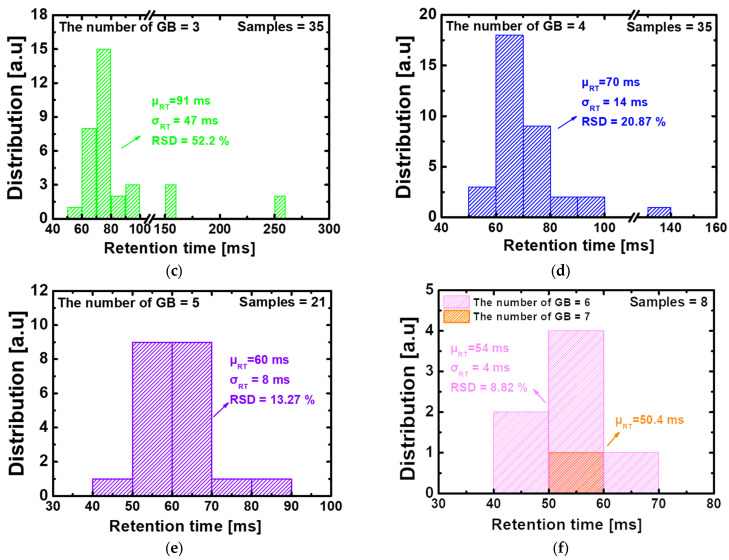
Histograms of the retention time for 127 samples in the proposed 1T-DRAM. (**a**) the number of GB is 1 (**b**) the number of GB is 2 (**c**) the number of GB is 3 (**d**) the number of GB is 4 (**e**) the number of GB is 5, and (**f**) the number of GB is 6 and 7.

Figure 11 shows the histogram of the retention time depending on the number of GBs. As can be seen from the previous sensing margin, the same trend is also observed in the retention time histogram graph. When the number of GB is one, the average maximum value is 223 ms, and the average minimum value is 54 ms when the number of GB is six. Accordingly, the maximum value of SD decreased from 144 ms to 4 ms and RSD also reduced from 64.53 ms to 8.82 ms. In particular, when the number of GB is five or higher, it can be seen that most of the samples exhibited retention times of 100 ms or less. As previously mentioned, the retention time is mainly affected when GB is located at B, F. When the number of GB is one, the probability that GB exists at B or F is 2/7 among all cases, and when the number of GB is two, the probability that GB exist at B or F is 11/21 among all cases. Therefore, the RSD increased when the number of GBs is two, compared to when the number of GBs is one. However, when the number of GB is three or more, the RSD gradually decreased because the probability that GB exists at B or F is larger than the probability that GB does not exist at B or F. The statistical analysis of the sensing margin and retention time depending on the number of GB is summarized in Table 6. When the number of GB is seven, there is one sample and only the average value exists. Although the retention times are affected by GBs, many samples are sufficient for retention time to meet 64 ms, which is the memory characteristic of the international roadmap for devices and systems (IRDS) [36].

## 4. Conclusions

In this study, a 1T-DRAM, based on a nanotube structure with GBs, is designed and simulated. GB forms a sharp energy band and prevents electrons flowing in the channel, which degraded the sensing margin and the retention time performance. When a single GB exists in the center of the body, it exhibits a superior sensing margin = 422 µA/µm and retention time = 213 ms. Additionally, considering the randomly generated GB, we analyzed the memory characteristics depending on the GB location and number in the proposed 1T-DRAM. The effect of GB on the sensing margin increased as the distance from the drain increased. Also, when the GB is located at the PN junction, the SRH recombination rate increases and the retention time degraded. In the worst case, we obtained the sensing margin = 398 µA/µm (when GB is located at C) and the retention time = 77 ms (when GB is located at F). In the case of the number of GB, mean, SD, and RSD variations of the sensing margin and retention time are studied. When the number of GB is seven, the mean of the sensing margin = 194 µA/µm and the mean of the retention time = 50.4 ms are obtained. When the number of GB is six, SD of the sensing margin is 31.1 μA/μm, and SD of the retention time is 4 ms. Compared with the single GB, the SD of the sensing margin and the retention time is reduced by 51% and 92%, respectively. In addition, RSD of the sensing margin decreased to 14.07% and RSD of the retention time decreased to 8.82%, respectively. Therefore, in the case of 1T-DRAM using poly–Si, the influence of GB cannot be ignored and should be considered (e.g., grain size, number, distribution, and location of GBs) for memory devices.

## Figures and Tables

**Table 1:** Geometric parameters of the proposed 1T-DRAM used for simulation.

Parameters	Values
Outer gate length (L_OG_)	50 nm
Inner gate length (L_IG_)	30 nm
Underlap length (L_underlap_)	10 nm
Body thickness (T_body_)	10 nm
Gate dielectric (HfO_2_) thickness (T_ox_)	2 nm
Source/Drain doping concentration	n-type 5 × 10^19^ cm^−3^
Body doping concentration	p-type 1 × 10^17^ cm^−3^
Outer gate work function	4.8 eV
Inner gate work function	5.2 eV

**Table 2:** Operating bias scheme of the proposed 1T-DRAM for memory performance.

Operation	Program (Write “1”)	Erase (Write “0”)	Read	Hold
Outer gate voltage [V]	2.0	0.0	1.0	0.0
Inner gate voltage [V]	−2.0	0.0	0.0	−0.5
Drain voltage [V]	0.0	−1.0	0.5	0.0

**Table 3:** Sensing margin and retention time depending on GB location of the proposed 1T-DRAM.

GB Location	A	B	C	D	E	F	G	w/o GB
Sensing margin [μA/μm]	536	458	398	422	458	547	554	556
Retention time [ms]	412	78	170	213	180	77	429	449

**Table 4:** Memory performance of various 1T-DRAM related papers.

No.	Reference	Sensing Margin [µA/µm]	Retention Time [ms]
1	[14]	2.51	598
2	[15]	14.10	251
3	[17]	0.39	10
4	[33]	11.7	148
5	[34]	2.5	33
6	[35]	12	170
7	This work (w/o GB)	422	213
8	This work (w/GB)	554	449

**Table 5:** Sample groups and number of samples from (A), (B), (C), (D), (E), (F), (G) to (A, B, C, D, E, F, G).

Groups	Number of Samples
(A), (B), (C), (D), (E), (F), (G)	7
(AB), (AC), (AD)…(FG)	21
(ABC), (ABD), (ABE)…(EFG)	35
(ABCD), (ABCE)…(DEFG)	35
(ABCDE), (ABCDF)…(CDEFG)	21
(ABCDEF), (ABCDEG)…(BCDEFG)	7
(ABCDEFG)	1

**Table 6:** Mean, standard deviation (SD), and relative standard deviation (RSD) of the sensing margin and the retention time depending on the number of GBs in the proposed 1T-DRAM.

	GB Number	1	2	3	4	5	6	7
	Mean	482	405	333	292	252	221	194
Sensing margin [μA/μm]	SD	63.5	94.4	85.8	63.4	46.9	31.1	X
	RSD	13.17%	23.27%	25.7%	21.68%	18.56%	14.07%	X
	Mean	223	127	91	70	60	54	50.4
Retention time [ms]	SD	144	92	47	14	8	4	X
	RSD	64.53%	72.09%	52.2%	20.87%	13.27%	8.82%	X

## Data Availability

Not applicable.

## References

[1] Kim K., Hwang C.-G., Lee J.G. (1998). DRAM technology perspective for gigabit era. IEEE Trans. Electron. Devices.

[2] Mandelman J.A., Dennard R.H., Bronner G.B., DeBrosse J.K., Divakaruni R., Li Y., Radens C.J. (2002). Challenges and future directions for the scaling of dynamic random-access memory (DRAM). IBM J. Res. Dev..

[3] Wann H.-J., Hu C. A capacitorless DRAM cell on SOI substrate. Proceedings of the IEEE International Electron Devices Meeting.

[4] Lee W., Choi W.Y. (2001). A novel capacitorless 1T DRAM cell for data retention time improvement. IEEE Trans. Nanotechnol..

[5] Yoshida E., Tanaka T. (2006). A capacitorless 1T-DRAM technology using gate-induced drain-leakage (GIDL) current for low-power and high-speed embedded memory. IEEE Trans. Electron. Devices.

[6] Song K.-W., Jeong H., Lee J.-W., Hong S.I., Tak N.-K., Kim Y.-T., Choi Y.L., Joo H.S., Kim S.H., Song H.J. 55 nm capacitor-less 1T DRAM cell transistor with non-overlap structure. Proceedings of the 2008 IEEE International Electron Devices Meeting.

[7] Giusi G., Iannaccone G. (2013). Junction engineering of 1T-DRAMs. IEEE Electron Dev. Lett..

[8] Giusi G., Alam M.A., Crupi F., Pierr S. (2010). Bipolar mode operation and scalability of double-gate capacitorless 1T-DRAM cells. IEEE Trans. Electron. Devices.

[9] Bawedin M., Cristoloveanu S., Flandre D. (2008). A capacitorless 1T-DRAM on SOI based on dynamic coupling and double-gate operation. IEEE Electron Dev. Lett..

[10] Kim S., Choi S.-J., Moon D.-I., Choi Y.-K. (2012). Carrier lifetime engineering for floating-body cell memory. IEEE Trans. Electron. Devices.

[11] Lee S.H., Park J., Kim G.U., Kang G.E., Heo J.H., Jeon S.R., Yoon Y.J., Seo J.H., Jang J., Bae J.-H. (2023). Bulk-fin field-effect transistor-based capacitorless dynamic random-access memory and its immunity to the work-function variation effect. Jpn. J. Appl. Phys..

[12] Lee S.H., Jang W.D., Yoon Y.J., Seo J.H., Mun H.J., Cho M.S., Jang J., Bae J.-H., Kang I.M. (2021). Polycrystalline-Silicon-MOSFET-Based Capacitorless DRAM With Grain Boundaries and Its Performances. IEEE Access.

[13] Lee S.H., Park J., Kim G.U., Min S.R., Jang J., Bae J.-H., Lee S.-H., Kang I.M. (2022). 3-D stacked polycrystalline-silicon-MOSFET-based capacitorless DRAM with superior immunity to grain-boundary’s influence. Sci. Rep..

[14] An H.D., Lee S.H., Park J., Min S.R., Kim G.U., Yoon Y.J., Seo J.H., Cho M.S., Jang J., Bae J.-H. (2022). De-sign of a Capacitorless DRAM Based on a Polycrystalline-Silicon Dual-Gate MOSFET with a Fin-Shaped Structure. Nanomaterials.

[15] Kim G.U., Yoon Y.J., Seo J.H., Lee S.H., Park J., Kang G.E., Heo J.H., Jang J., Bae J.-H., Lee S.-H. (2022). Design of a Capacitorless DRAM Based on Storage Layer Separated Using Separation Oxide and Polycrystalline Silicon. Electronics.

[16] Park J., Cho M.S., Lee S.H., An H.D., Min S.R., Kim G.U., Yoon Y.J., Seo J.H., Lee S.-H., Jang J. (2021). Design of Capacitorless DRAM Based on Polycrystalline Silicon Nanotube Structure. IEEE Access.

[17] Yoon Y.J., Lee J.S., Kim D.-S., Lee S.H., Kang I.M. (2020). One-transistor dynamic random-access memory based on gate-all-around junction-less field-effect transistor with a Si/SiGe heterostructure. Electronics.

[18] Yoon Y.J., Seo J.H., Cho S., Lee J.-H., Kang I.M. (2019). A polycrystalline-silicon dual-gate MOSFET-based 1T-DRAM using grain boundary-induced variable resistance. Appl. Phys. Lett..

[19] Kim H., Yoo S., Kang I.M., Cho S., Sun W., Shin H. (2020). Analysis of the Sensing Margin of Silicon and Poly-Si 1T-DRAM. Micromahines.

[20] (2016). Sentaurus Device User Guide Version L-2016.03.

[21] Jang W.D., Yoon Y.J., Cho M.S., Jung J.H., Lee S.H., Jang J., Bae J.-H., Kang I.M. (2020). Polycrystalline silicon metal-oxide-semiconductor field-effect transistor-based stacked multi-layer one-transistor dynamic random-access memory with double-gate structure for the embedded systems. Jpn. J. Appl. Phys..

[22] Hanna N., Hussain M.M. (2015). Si/Ge hetero-structure nanotube tunnel field effect transistor. J. Appl. Phys..

[23] Sahay S., Kuma M.J. (2017). Nanotube junctionless FET: Proposal, design, and investigation. IEEE Trans. Electron. Devices.

[24] Tekleab D., Tran H.H., Slight J.W., Chidambarrao D. (2012). Silicon Nanotube MOSFET. U.S. Patent.

[25] Fahad H.M., Smith C.E., Rojas J.P., Hussain M.M. (2011). Silicon nanotube field effect transistor with core–shell gate stacks for enhanced high-performance operation and area scaling benefits. Nano Lett..

[26] Tiwari P.K., Samoju V.R., Sunkara T., Dubey S., Jit S. (2016). Analytical modeling of threshold voltage for symmetrical silicon nano-tube field-effect-transistors (Si-NT FETs). J. Comput. Electron..

[27] Ho C.H., Panagopoulos G., Roy K. (2012). A Physical Model for Grain-Boundary-Induced Threshold Voltage Variation in Polysilicon Thin-Film Transistors. IEEE Trans. Electron. Devices.

[28] Ranica R., Villaret A., Fenouillet-Beranger C., Malinge P., Mazoyer P., Masson P., Delille D., Charbuillet C., Candelier P., Skotnicki T. A capacitor-less DRAM cell on 75 nm gate length, 16 nm thin Fully Depleted SOI device for high density embedded memories. Proceedings of the IEDM Technical Digest. IEEE International Electron Devices Meeting.

[29] Yoon S., Kim K., Cho H., Yoon J.-S., Lee M.J., Meyyappan M., Baek C.-K. (2017). Polysilicon near-infrared photodetector with performance comparable to crystalline silicon devices. Opt. Express.

[30] Kimura M., Inoue S., Shimoda T., Sameshima T. (2001). Device simulation of carrier transport through grain boundaries in lightly doped polysilicon films and dependence on dopant density. Jpn. J. Appl. Phys..

[31] Troutman R.R. (1979). VLSI limitations from drain-induced barrier lowering. IEEE J. Solid-State Circuits.

[32] Sajjad R.N., Chern W., Hoyt J.L., Antoniadis D.A. (2016). Trap assisted tunneling and its effect on subthreshold swing of tunnel FETs. IEEE Trans. Electron. Devices.

[33] Ansari MH R., Cho S. (2021). Performance Improvement of 1T DRAM by Raised Source and Drain Engineering. IEEE Trans. Electron. Devices.

[34] Ansari M.H.R., Navlakha N., Lee J.Y., Cho S. (2020). Double-gate junctionless 1T DRAM with physical barriers for retention improvement. IEEE Trans. Electron. Devices.

[35] James A., Saurabh S. (2019). Dopingless 1T DRAM: Proposal, design, and analysis. IEEE Access.

[36] (2021). More Moore, 2021 International Roadmap for Devices and Systems (IRDS™) Edition. https://irds.ieee.org/editions/2021.

